# Managing Lead Fractures in Deep Brain Stimulation for Movement Disorders: A Decade-Long Case Series from a National Neurosurgical Centre

**DOI:** 10.3390/jcm13247509

**Published:** 2024-12-10

**Authors:** Chingiz Nurimanov, Iroda Mammadinova, Karashash Menlibayeva, Seitzhan Aidarov, Nurtay Nurakay, Assylbek Kaliyev, Yerbol Makhambetov, Serik K. Akshulakov

**Affiliations:** 1 Department of Vascular and Functional Neurosurgery, National Centre for Neurosurgery, 34/1 Turan Avenue, Astana 010000, Kazakhstan; 2 Hospital Management Department, National Centre for Neurosurgery, 34/1 Turan Avenue, Astana 010000, Kazakhstan

**Keywords:** deep brain stimulation, hardware-related complication, lead fractures, extension failure

## Abstract

**Background**: Deep brain stimulation (DBS) is an effective treatment for movement disorders, but its long-term efficacy may be undermined by hardware complications such as lead fractures. These complications increase healthcare costs and necessitate surgical revisions. The frequency, timing, and clinical factors associated with lead fractures remain poorly understood. **Objective:** This study aimed to determine the incidence, timing, and clinical factors associated with lead fractures in a large cohort of DBS patients over a 10-year period. **Methods**: This retrospective study analyzed data from 325 patients who underwent bilateral DBS implantation at the National Centre for Neurosurgery from 2013 to 2023. The analysis specifically focused on 17 patients who experienced lead fractures during the long-term follow-up period. **Results**: Among the 325 patients, lead fractures were identified in 17 patients (5.23%), affecting 18 electrodes. The majority of cases involved patients with Parkinson’s disease (76.5%) or dystonia (23.5%), with an average age of 59.17 ± 8.77 years. Nearly all patients with lead fractures had a history of trauma. Additionally, two cases were associated with active engagement in sports, particularly activities involving movements like pulling up on a horizontal bar, while Twiddler’s Syndrome was identified in two other cases. All electrode fractures required surgical revision. **Conclusions**: Lead fractures, while rare, remain a significant complication in DBS systems. Precise surgical techniques, early detection, and advancements in DBS hardware design may help to mitigate this risk. Future innovations, such as durable leads or wireless systems, may improve long-term outcomes in DBS therapy for movement disorders.

## 1. Introduction

Since its introduction in the 1980s, deep brain stimulation (DBS) has largely replaced ablative procedures like thalamotomy, pallidotomy, and subthalamotomy due to its safety, reversibility, and lower rates of neurological morbidity and mortality [1,2]. These benefits make DBS a preferred treatment option for movement disorders, including Parkinson’s disease, essential tremor (ET), and dystonia [3,4,5]. Over the past two decades, its clinical applications have expanded to cover a range of neurological and psychiatric conditions, such as Tourette’s syndrome, refractory epilepsy, and obsessive–compulsive disorder [6].

Despite its advantages reversible procedure, DBS has certain drawbacks. These include high costs, the need for frequent follow-up, eventual battery replacements, and potential device-related complications [7,8].

The complications associated with DBS can be categorized into main groups: procedural, device-related malfunction, stimulation-related complications, and other patient complaints [9,10]. A Manufacturer and User Facility Device Experience (MAUDE) database analysis identified infection as the most common surgical complication (36.4%) and lead migration as the most frequent device-related issue (38.8%), followed by device failures (28.6%), impedance problems (22.4%), and lead fractures (10.2%). Neurostimulation-related symptoms included inadequate stimulation, worsening Parkinson’s disease symptoms, seizures, and incision site burning [10]. In addition, DBS is associated with potential side effects, including cognitive decline. Despite its efficacy in improving motor symptoms, some patients may experience impairments in verbal fluency, executive function, and memory [11].

A DBS system consists of several components, and malfunctions in these components can lead to hardware-related complications. Prevalent complications including lead migration, lead fractures or failures, implantable pulse generator (IPG) malfunctions, and more device-related concerns [9,12]. Lead fractures, the frequent hardware-related complication, vary between 1% and 15.2% in different series and the factors are not well understood [13,14], requiring surgical revision, compromising long-term benefits and increasing healthcare costs [15].

This study aimed to determine the frequency and timing of wire-related complications, particularly lead fractures, as well as associated clinical factors in a large series of patients with movement disorders treated with DBS over a decade at a national neurosurgical centre.

## 2. Materials and Methods

This retrospective study analyzed a consecutive series of patients who underwent DBS surgery at the National Centre for Neurosurgery from 2013 to 2023 to investigate the long-term incidence of lead fractures. The same team of surgeons, utilizing uniform surgical techniques and equipment, performed all surgical procedures.

Overall, 325 patients were included in the study, all of whom received bilateral DBS implants (Medtronic (Medtronic Inc., Minneapolis, MN, USA), Scene Ray (SceneRay Co., Ltd., Suzhou, China) (11 patients)). The primary diagnoses among the patients were Parkinson’s disease, dystonia, ET, and Tourette syndrome.

The DBS system consisted of an implantable pulse generator, an extension electrode, and a stimulation electrode. The DBS electrodes were surgically implanted in the internal pallidus (GPi), subthalamic nucleus regions (STN), and ventral intermediate nucleus (VIM). The connection point between the DBS electrode and the extension cable was consistently located in the left mastoid region for all cases. The extension electrode served to connect the IPG to the lead subcutaneously. The IPG was implanted either subcutaneously or submuscularly in the infraclavicular region. It was fixed to the fascia of the major pectoral muscle using sutures. All electrode and IPG implantations were completed during a single surgical session at our center.

Besides demographic information, the database also recorded neurological diagnoses, surgical targets, implantation dates, follow-up periods, clinical symptoms associated with complications, and revisions to the implanted DBS system.

For retrospective analysis, the study period was determined to allow a minimum follow-up of 6 months. The study specifically focused on 17 patients who underwent DBS surgery and subsequently experienced lead fractures during the long-term follow-up period. Patients who were lost to follow-up were excluded from the study.

## 3. Results

Our study encompassed 325 patients, undergoing 652 electrode implantations across 325 surgical procedures from June 2013 to September 2023. The participant diagnoses were primarily Parkinson’s disease in 274 patients (84.30%), followed by dystonia in 45 patients (13.84%), ET in 5 patients (1.53%), and 1 patient (0.3%) with Tourette syndrome. The electrode target distribution included the VIM of the thalamus in six cases (1.84%), the STN in 271 cases (83.38%), and the GPi in 48 cases (14.76%).

Over the follow-up period, concluding in April 2024 with a minimum duration of 6 months, we observed lead fractures in 17 of the 325 patients (5.23%). These incidents involved a total of 18 electrodes. The demographic characteristics of the affected patients are detailed in Table 1. The average age at the time of DBS implantation in this subgroup was 59.17 ± 8.77 years, ranging from 32 to 72 years. The distribution among genders consisted of nine females and eight males. Among the patients experiencing lead fractures, 13 were diagnosed with Parkinson’s disease at stages 3 and 4, while four patients had dystonia.

Lead fractures were predominantly identified through clinical presentations and corroborated by high impedances associated with low currents in 2–4 contacts of the same electrode, leading to a precipitous loss of the DBS therapeutic effect.

Diagnostic procedures, including X-rays, confirmed the presence of complete fractures in three patients and a kinked electrode indicative of “Twiddler’s syndrome” in two patients. In 11 patients, no complete fracture was diagnosed via X-ray; however, examination revealed high impedance levels (>2000) and low currents (<9 A) across all contacts, suggesting internal lead damage. Notably, in one case where both impedance levels and X-ray results appeared normal without clinical effect on stimulation, structural changes in the lead were observed, although not amounting to a complete fracture.

The localization of lead fractures varied, with 11 cases presenting below the clavicle, and four fractures above the clavicle (neck region). One patient had a cranial lead fracture, and one patient had a fracture in the connection point between the lead and extension.

Interestingly, almost all patients with lead fractures reported a history of trauma, whereas two cases were linked to active participation in sports, specifically involving movements such as pulling up on a horizontal bar, and, in two cases, Twiddler’s Syndrome was observed. In all cases, management was involved in replacing the extension wires, which successfully restored normal impedance levels. Following these interventions, 100% of patients regained the clinical benefits of DBS.

**Figure 1:**
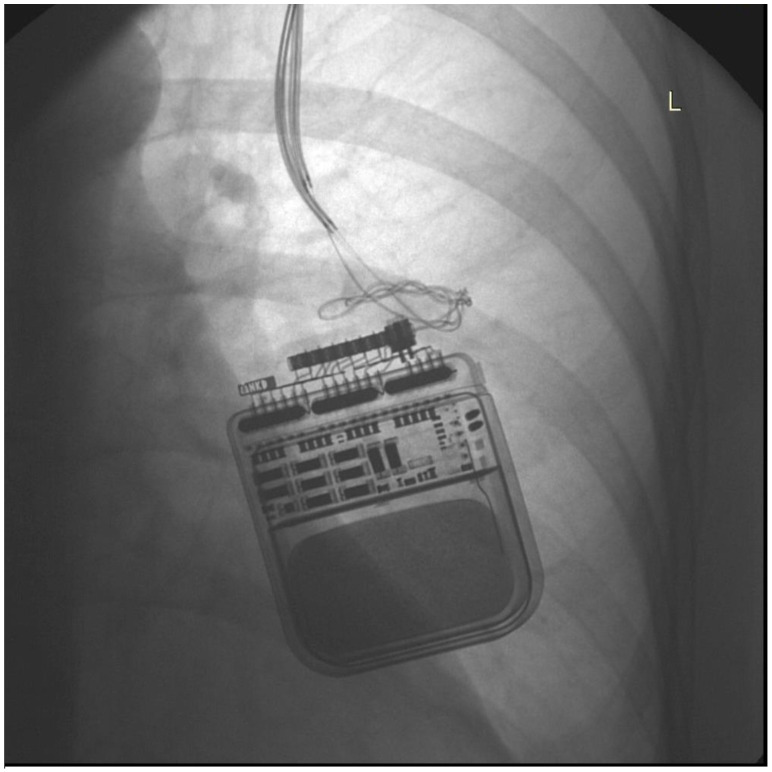
Chest X-ray showing IPG and coiling of extension wires.

Illustrative case 1. Patient 11, is a 55-year-old female patient diagnosed with multifocal dystonia, manifesting as cervical and torsion dystonia, for the past 11 years. This patient achieved significant symptomatic improvement following bilateral GPi DBS and the implantation of a left dual-channel IPG (Activa PC), a treatment modality that has been effectively managing her condition for ten years. Battery replacements for the IPG were necessitated 5.5 years and again 3.5 years before the most recent procedure, due to the exhaustion of battery capacity. The patient was readmitted to the hospital, presenting with worsened uncontrolled muscle cramps and spasms, as well as difficulties in speaking and swallowing, symptoms suggestive of an intensification of her dystonia. Upon evaluation, impedance and current measurements for electrodes 0, 1, and 2 were within normal ranges, suggesting intact circuitry. However, electrode 3 displayed significantly elevated impedance levels (10,700–12,400) and diminished current output (0.6 mA), indicating a potential lead malfunction. Subsequent chest X-rays (Figure 1) revealed pronounced coiling of the DBS extension leads, a mechanical complication that could compromise DBS efficacy. An emergency surgical revision was necessitated. During the procedure, twisting of the DBS extension leads was observed, necessitating replacement. Additionally, the IPG was securely repositioned beneath the pectoralis fascia to mitigate future displacement and coiling risks. This intervention significantly improved the patient’s dystonic symptoms, with follow-up examinations revealing normalization of impedance levels.

**Figure 2:**
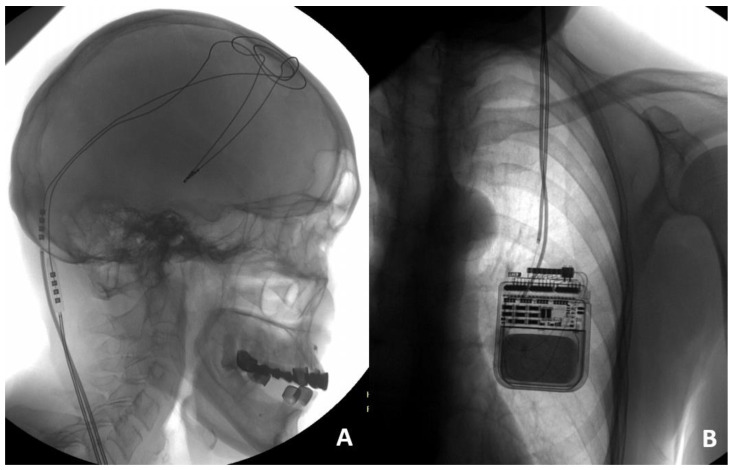
Skull X-ray illustrating intact DBS leads (**A**). Chest X-ray showing the lead fracture with connection located in subclavicular region (**B**).

Illustrative case 2. Patient 12, a 63-year-old female diagnosed with stage 3 Parkinson’s disease according to the Hoehn and Yahr scale, has been managing her condition with bilateral STN DBS using a left dual-channel IPG (Activa PC) for two years. The initial programming of the DBS system yielded significant symptomatic improvement. Recently, the patient experienced a gradual worsening of her condition, notably two weeks following a fall. This decline in her condition necessitated a thorough evaluation to examine the functionality of the DBS system. Electrode impedance measurements for electrodes 8 through 10 were found to exceed 2000 Ω, suggesting a potential malfunction of the lead system. To ascertain the cause of the suspected malfunction, the patient underwent diagnostic imaging, including cranial and chest X-rays. The cranial and chest X-rays revealed no abnormalities in the hardware configuration (Figure 2A,B), suggesting that all DBS system components remained intact. However, due to acute worsening of the patient’s condition and lack of stimulation effect, a decision was made to revise the extensions, and an intraoperative incomplete left extension fracture in the neck region was found. After the diagnostic findings, surgical intervention was undertaken to replace the malfunctioning left extension wires. This corrective measure successfully restored the efficacy of the DBS therapy, evidenced by the patient regaining control over her tremors.

**Figure 3:**
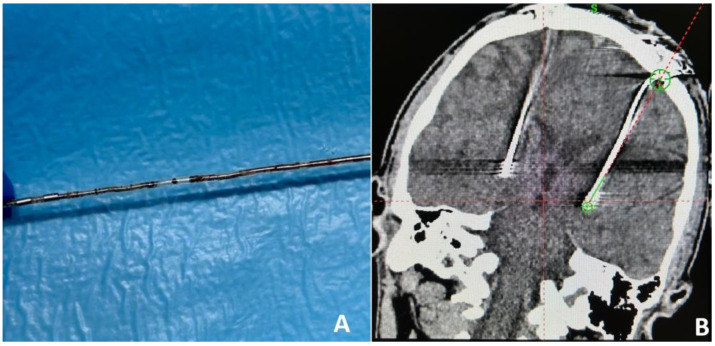
Intracranial extension wire fracture (**A**). Post-operative frontal CT after intracranial electrode replacement (**B**).

Illustrative case 3. Patient 9, a 32-year-old male with a 13-year history of the condition, had previously experienced significant symptomatic relief from bilateral GPi DBS and IPG (Activa PC) therapy for eight years. A routine replacement of the IPG was performed one year prior due to battery capacity exhaustion. The patient presented to the medical facility with acute involuntary muscle contractions in the neck, a symptom onset that followed physical exertion from pull-ups. This event prompted an urgent reassessment of the DBS system’s functionality. Initial impedance and current measurements indicated functional discrepancies across the electrodes: electrode 2 exhibited normal parameters, whereas electrodes 0, 1, and 3 showed elevated impedance levels (3946–4278) and reduced current (0.7 μA), suggesting the presence of three open circuits. Comprehensive cranial and chest radiographic examinations failed to reveal any structural anomalies within the hardware. An initial decision to revise the extension wires was made based on the diagnostic findings. However, this intervention did not yield an improvement in the patient’s symptoms. Further impedance assessments revealed persistently high levels across electrodes 0–3. Subsequent surgical exploration revealed a lead fracture at the junction between the intracranial electrode and its extension (Figure 3A), which was determined to be the underlying cause of the system’s malfunction. The patient underwent a successful stereotactic reimplantation of the left intracranial electrode and extension. Post-operative computed tomography (CT) imaging (Figure 3B) confirmed the precise placement of the newly implanted electrode within the targeted GPi region. The post-surgical period was marked by a notable improvement in the patient’s symptoms, aligning with the normalization of impedance levels across the evaluated electrodes.

**Figure 4:**
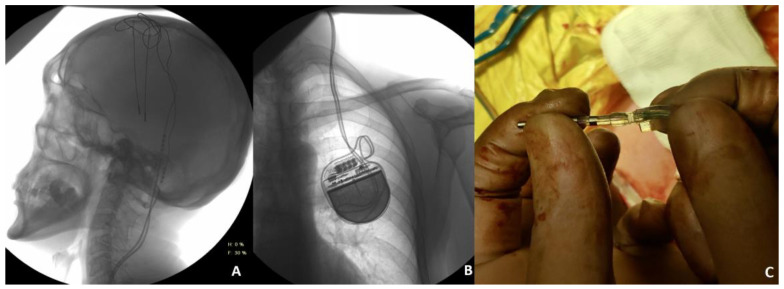
Skull (**A**) X-ray showing any deformities of the leads. Chest (**B**) X-ray showing the reversed position of the extensions at the junction connecting to the IPG. Intraoperative view of the lead fracture at the point of connection between extension and IPG (**C**).

Illustrative case 4. Patient 14, a 57-year-old male diagnosed with Parkinson’s disease at stage 3 according to the Hoehn and Yahr scale, had been managing his condition effectively for eight years. The patient had undergone bilateral STN DBS with IPG (Scene Ray Aaxon) for one year, witnessing sustained improvement in motor symptoms. Stability in motor function was abruptly compromised following a trauma incurred during physical exercise, specifically pull-ups. This incident prompted an immediate evaluation of the DBS system to ascertain the impact of the trauma on its functionality. Initial impedance and current measurements identified an open circuit condition in the electrodes targeting the left STN, characterized by impedance levels exceeding 2000 Ω and current measurements below 7 μA. Conversely, electrodes positioned elsewhere displayed normal operational metrics. Radiographic imaging of the chest and skull, aimed at identifying potential hardware deformities, yielded negative results (illustrated in Figure 4A,B), suggesting no visible structural damage to the system components. Given the diagnostic findings and the acute onset of symptoms post-trauma, an emergent surgical revision was deemed necessary. During the procedure, a fracture in the left DBS extension lead was identified at the junction connecting the extension wire to the pulse generator (Figure 4C). This mechanical failure was pinpointed as the critical factor undermining the system’s efficacy. The replacement of the left extension wire was performed, resulting in a significant improvement in the patient’s motor symptoms during subsequent follow-ups.

## 4. Discussion

Deep brain stimulation, while highly effective, as any surgical procedure, carries inherent risks and potential complications. Among these, hardware-related issues—particularly lead fractures—can significantly impact the postoperative course of patients with DBS implants. These complications may interrupt the continuous DBS therapy essential for managing movement disorders, leading to adverse outcomes and diminished therapeutic efficacy. In severe cases, the removal and replacement of hardware become necessary, increasing procedural costs and reducing overall cost-effectiveness [16,17].

This study investigates the prevalence of hardware-related complications, focusing on lead fractures, over 10 years. Lead fractures represent the third most common hardware-related complication, with reported incidence rates ranging from 1.46% to 15.2% in the literature, underscoring their clinical significance [12,14,18]. At our institution, lead fractures were observed in 2.76% of implanted wires, affecting 5.23% of patients who underwent DBS during the study period. These findings highlight the importance of identifying and addressing hardware-related complications to optimize patient outcomes and maintain the long-term efficacy of DBS therapy.

The reasons that contribute to lead fracture or tethering remain unknown. Trauma, impulsive neck jerks, flexion, rotation, and spontaneous events have all been considered probable causes of lead fractures or failure. In addition, mechanical stress on the lead, improper alignment, and excessive or inadequate movement can all contribute to lead fractures [12,19]. In a study by Jitkritsadakul et al., it was found that the highest rate of lead fracture or failure was observed in dystonia patients, with a rate of 4.22% [12]. This finding suggests a potential association between abnormal movements of the neck and lead fractures.

Dystonia patients may experience constant stress on the lead due to their neck movements in daily life, such as bending, tilting, or twisting. It is suspected that these movements transmit varying stress to the lead, leading to its weakening and eventual breakage [18,19,20]. However, in our study, only four patients with dystonia experienced lead fractures. After DBS treatment, they no longer exhibited the typical stereotyped movements of dystonia. The fractures were caused by trauma during sports activities and falls. In our study, we found that abruption was significantly more associated with falls than with sports or spontaneous disruption. Our research revealed that lead fractures were observed in patients with Parkinson’s disease in stages 3–4, indicating a higher risk of falling due to postural instability disorders [21]. Conversely, a study by Blomstedt et al. found that 7 out of 8 electrode fractures occurred in patients with ET, suggesting that head tremors may have contributed to the fractures in some cases [15].

The occurrence of lead fractures can compromise the therapeutic efficacy of DBS, leading to an abrupt worsening of symptoms and potentially necessitating hardware revision. Clinically, lead fractures present a diagnostic challenge due to the varied and often nonspecific nature of symptom recurrence. In our case series, the primary clinical manifestation was the reappearance of movement disorder symptoms, such as tremors or rigidity, reflecting the loss of DBS efficacy. In several cases, there was a delay in diagnosis due to the gradual and intermittent nature of the symptoms worsening. In all cases analyzed in our study, patients exhibited a sudden worsening of their condition. When lead fracture is suspected, it is essential to assess electrode impedance, evaluate voltage-related side effects, and conduct a radiological examination [19]. In some cases, variations in impedance and current measurements with different body positions, often linked to intermittent stimulation patterns, may be due to microfractures allowing temporary connections between the open circuit’s two poles during changes in neck or head positions [22,23]. On the other hand, it can be argued that in cases of elevated impedance levels, normal lead might be observed on the X-ray, particularly in situations involving incomplete fractures or microfractures. These are typically only detectable during intraoperative examinations.

Implementing careful techniques when handling the extension wire and its connections, along with allowing some flexibility for movement, has proven effective in minimizing complications. An experimental study performed by Jiang C et al. emphasizes that maintaining the integrity of the wire’s helical structure is essential for the lead’s fatigue performance [24]. The electrodes contain platinum wires that are vulnerable to being crushed by screws. Any part of the electrode exposed to stretching or compression is at risk of complications, with most fractures typically occurring near the connection between the lead and the extension [25]. Mackel et al. reported that connections between the lead and the extension cable in the cervical region are linked to an increased risk of wire fractures. Therefore, the use of cervical connectors should be approached with caution due to the potential risk of lead wire fracture [26]. Fernández et al. found that the most common site for electrode cable breakage was typically located between 9 and 13 mm from the junction between the lead and the extension cable [19]. According to Constantoyannis C et al., placing the connection to the lead extender more superiorly in the parietal regions, rather than in a post-auricular area, may reduce the risk of lead fracture [25]. We support this statement, suggesting that this approach potentially minimizes the transmission of severe cervical traction to the lead.

The extension cable is less likely to break when twisted due to its greater resilience, whereas the electrodes, being more delicate and anchored to the cranium, are more susceptible to the forces from these movements [22]. Twiddler syndrome (twisted extension cables) is more common in patients with cardiac implantable electronic devices (CIED). It involves multiple rotations of the IPG within the subcutaneous pocket, often accompanied by coiling of the electrodes. This can result in system malfunction due to electrode breakage or displacement. Clinically, a hypermobile stimulator and thickened cable strands or pulls can be felt under the skin [27]. For Patients 11 and 17, encountering twisted leads following DBS implantation presented a complex issue that demanded immediate identification and action to regain the therapeutic benefits of the DBS system. The exact cause of Twiddler’s Syndrome in these cases remains unclear; however, in all instances, the IPG was secured using two silk sutures through the anchor hole to the muscle fascia. In such cases, some experts suggest employing the “Dual Anchor Internal Pulse Generator Technique”, which involves additional suturing to the overlying subcutaneous tissue, or using an absorbable Antibacterial Envelope [27,28].

While previous studies, such as Mackel CE et al., have suggested potential risks associated with different wire lengths [26], our use of exclusively 40 cm wires precluded a direct comparison with 60 cm wires. We also advocate against connecting the lead and the extension cable in the cervical regions to minimize associated risks [26]. Additionally, we recommend creating a wide loop in the lead at the cranial level to prevent electrode displacement and advise leaving a reserve length of the extensions in the IPG pocket and above the steam locks. During IPG implantation, we recommend rotating the head by 65–70 degrees in the opposite direction to avoid cervical tethering and maintain cervical movement freedom. We suggest securing the IPG with silk sutures through its two anchor holes to the muscle fascia to prevent migration or twisting of the extension. In instances of cranial electrode fracture, successful management may be achieved through the implantation of a new electrode, as demonstrated in the case of Patient 9.

The proper positioning of all components in a DBS system is crucial for its optimal functioning and to minimize potential complications. Manufacturers may have specific guidelines or recommendations for the positioning of system components, including the extensions and the IPG itself. A key advantage of adhering to these guidelines is the ability to quickly diagnose any malposition of the DBS components using X-ray imaging. In our case series, we observed a misalignment of the extensions at the connection junction to the IPG in Patient 14. Regular monitoring of the system and stimulation parameters is essential for the timely diagnosis of hardware-related complications and disease progression in those patients.

Detecting lead fractures poses a significant challenge. While complete lead fractures can be identified through X-ray imaging, micro-fractures—another potential cause of DBS system failure—are often undetectable, even with impedance measurements [29]. This necessitates a systematic approach, starting with checking the IPG side of the extensions, followed by inspecting the connection site between the extension wire and the intracranial lead in the postauricular region. These additional steps increase the duration of the procedure and elevate the risk of infection.

Surgical interventions used to address complications in DBS cases include removing and replacing hardware, implanting rescue leads, and revising wounds [10,30]. Hardware removal is typically indicated in cases of severe infection. Replacement of DBS leads is necessary when the existing lead is in a suboptimal position, has fractured, or is ineffective despite proper placement [7,31]. In our case series, all patients with diagnosed lead fractures underwent extension wire replacement. The procedure began with the revision of the integrity of the extension wires at the IPG site. A skin incision was then made in the postauricular region to access the extension wires. Following the replacement of the extension wire, impedance was measured. If high impedance was detected, an intracranial replacement procedure was performed to ensure proper functionality. In cases requiring intracranial lead replacement, the procedure becomes even more complex. It typically requires a second surgery using a stereotactic frame and the acquisition of a new MRI for precise targeting. This approach is not only time-consuming and economically burdensome but also imposes additional stress on the patient.

Across our cohort, there were no significant variations in acute reactions to the surgical procedures. All patients tolerated the interventions well, with no immediate complications such as infections, hematoma formation, or hardware-related issues observed during the post-operative period. The only observed reactions were localized pain and mild swelling at the surgical site, which resolved within a few days without requiring additional interventions. However, we acknowledge that the absence of significant acute reactions in our study may not fully reflect broader clinical experiences, and further studies with larger patient groups could help confirm these results.

Patients with lead fractures who underwent extension replacement received extended antibacterial therapy for 5–7 days, compared to the standard 3-day course typically prescribed for other DBS patients in our clinic. Hospital stay duration was also prolonged for these patients due to the need for additional adjustments to stimulation parameters following the extension replacement, tailored to their neurological condition.

Before discharge, patients were provided with additional education on safety guidelines specifically for those with DBS systems to help prevent future complications. After DBS implantation, patients often experience improvements in their quality of life and activity levels. Our case series suggests that increased activity levels, including excessive head, shoulder, and arm extensions, as well as rotations during sports and physical activities, may elevate the risk of hardware fractures. Such movements can exert additional stress on the DBS system components, potentially compromising their structural integrity over time. Given these findings, it is crucial to advise patients to exercise caution and avoid activities involving repetitive or forceful movements of the head, shoulders, and arms following DBS implantation. Patient education and awareness about the potential risks associated with certain activities post-DBS are essential for minimizing complications. Future advancements in technology and improvements in DBS devices are expected to further reduce hardware-related complications, particularly with the development of wireless systems.

### Limitations

The study was conducted on patients treated in a specialized neurosurgical hospital, which may limit the generalizability of the findings to the broader population. Additionally, this selection could introduce discrepancies between the study data and outcomes observed in routine clinical practice.

## 5. Conclusions

Lead fractures remain a rare but significant complication in DBS systems, particularly during long-term follow-up. Our results indicate that over 10 years at our center, lead fractures occurred in 5.23% of the patients who received DBS. Our findings underscore the importance of early recognition, precise surgical technique, and potential preventive measures to mitigate this risk. Future technological advancements, such as more durable leads or wireless systems, may further reduce the incidence of this complication, enhancing the longevity and efficacy of DBS therapy for movement disorders.

## Figures and Tables

**Table 1:** Patient characteristics.

Patients	Patients Age, Year/Sex	Diagnosis	Place of Implantation DBS	Date of Implant	IPG Replacement Procedures	Date of Revision Surgery (dd/mm/yyyy)	Place of Break (Intraoperative)	Causes	Impedance, Ω	Side of Fracture
1	59 F	Parkinson’s Disease stage 4	STN	28 November 2013	2	22 November 2019	infraclavicular region	trauma	23,986–24,171	Right
2	67 F	Parkinson’s Disease stage 4	STN	14 September 2016	2	22 June 2020	neck	trauma	13,120–15,505	Left
3	48 M	Cervical dystonia	GPI	24 November 2017	-	24 November 2022	neck	trauma	normal	Both
4	64 M	Parkinson’s Disease stage 4	STN	30 June 2015	1	2 September 2022	connection between the lead and extension	trauma	4612–5481	Right
5	58 M	Parkinson’s Disease stage 3	STN	21 April 2023	-	29 December 2023	infraclavicular region	trauma	3010–3121	Left
6	68 F	Parkinson’s Disease stage 4	STN	4 April 2018	1	16 May 2023	infraclavicular region	trauma	2477–2495	Right
7	59 F	Parkinson’s Disease stage 4	STN	12 February 2020	1	2 March 2023	infraclavicular region	trauma	12,185–12,942	Right
8	61 M	Dystonia	GPI	28 April 2021	-	26 October 2023	neck	NA	2518–3423	Left
9	32 M	Cervical dystonia	GPI	6 October 2015	-	12 December 2023	head	active sport	3946–4278	Left
10	72 F	Parkinson’s Disease stage 5	STN	3 August 2017	1	24 January 2024	neck	trauma	High	Right
11	55 F	Dystonia	GPI	21 November 2014	-	25 January 2024	infraclavicular region	Twiddler’s Syndrome	10,700–12,400	Left
12	63 F	Parkinson’s Disease stage 3	STN	5 August 2022	-	6 February 2024	infraclavicular region	trauma	2388–2688	Right
13	57 M	Parkinson’s Disease stage 3	STN	20 April 2022	-	20 September 2023	infraclavicular region	trauma	3243–3962	Left
14	57 M	Parkinson’s Disease stage 3	STN	26 August 2021	-	22 January 2024	connection between the extensions and IPG	active sport	High	Left
15	57 F	Parkinson’s Disease stage 4	STN	2014	2	27 December 2023	infraclavicular region	trauma	5496–6540	Left
16	62 F	Parkinson’s Disease stage 4	STN	24 May 2021	-	20 February 2024	infraclavicular region	trauma	High	Left
17	67 M	Parkinson’s Disease stage 3	STN	26 August 2021	-	3 April 2024	infraclavicular region	Twiddler’s Syndrome	High	Left

Abbreviations: F—female, M—male, DBS—deep brain stimulation, STN—Subthalamic nucleus, GPI—Internal globus pallidus, NA—not available.

## Data Availability

Data are contained within the article.
